# Imputation of orofacial clefting data identifies novel risk loci and sheds light on the genetic background of cleft lip ± cleft palate and cleft palate only

**DOI:** 10.1093/hmg/ddx012

**Published:** 2017-01-19

**Authors:** Kerstin U. Ludwig, Anne C. Böhmer, John Bowes, Miloš Nikolić, Nina Ishorst, Niki Wyatt, Nigel L. Hammond, Lina Gölz, Frederic Thieme, Sandra Barth, Hannah Schuenke, Johanna Klamt, Malte Spielmann, Khalid Aldhorae, Augusto Rojas-Martinez, Markus M. Nöthen, Alvaro Rada-Iglesias, Michael J. Dixon, Michael Knapp, Elisabeth Mangold

**Affiliations:** 1 Institute of Human Genetics University of Bonn, Bonn 53127, Germany; 2 Department of Genomics, Life and Brain Center, University of Bonn, Bonn 53127, Germany; 3 Arthritis Research UK Centre for Genetics and Genomics, The University of Manchester, Manchester M13 9PT, UK; 4 Center for Molecular Medicine Cologne; 5 Cologne Excellence Cluster for Cellular Stress Responses in Aging-Associated Diseases (CECAD), University of Cologne, Cologne 50931, Germany; 6 Faculty of Biology, Medicine and Health, University of Manchester, Manchester M13 9PT, UK; 7 Department of Orthodontics, University of Bonn, Bonn 53111, Germany; 8 Max Planck Institute for Molecular Genetics, RG Development and Disease, Berlin 14195, Germany; 9 Institute for Medical and Human Genetics; 10 Berlin-Brandenburg Center for Regenerative Therapies (BCRT), Charité Universitätsmedizin Berlin, Berlin 13353, Germany; 11 Orthodontic Department, College of Dentistry, Thamar University, Thamar, Yemen; 12 Tecnologico de Monterrey, School of Medicine, and Universidad Autonoma de Nuevo Leon, Centro de Investigación y Desarrollo en Ciencias de la Salud, Monterrey 64460, Mexico; 13 Institute of Medical Biometry, Informatics and Epidemiology, University of Bonn, Bonn 53127, Germany

## Abstract

Nonsyndromic cleft lip with or without cleft palate (nsCL/P) is among the most common human birth defects with multifactorial etiology. Here, we present results from a genome-wide imputation study of nsCL/P in which, after adding replication cohort data, four novel risk loci for nsCL/P are identified (at chromosomal regions 2p21, 14q22, 15q24 and 19p13). On a systematic level, we show that the association signals within this high-density dataset are enriched in functionally-relevant genomic regions that are active in both human neural crest cells (hNCC) and mouse embryonic craniofacial tissue. This enrichment is also detectable in hNCC regions primed for later activity. Using GCTA analyses, we suggest that 30% of the estimated variance in risk for nsCL/P in the European population can be attributed to common variants, with 25.5% contributed to by the 24 risk loci known to date. For each of these, we identify credible SNPs using a Bayesian refinement approach, with two loci harbouring only one probable causal variant. Finally, we demonstrate that there is no polygenic component of nsCL/P detectable that is shared with nonsyndromic cleft palate only (nsCPO). Our data suggest that, while common variants are strongly contributing to risk for nsCL/P, they do not seem to be involved in nsCPO which might be more often caused by rare deleterious variants. Our study generates novel insights into both nsCL/P and nsCPO etiology and provides a systematic framework for research into craniofacial development and malformation.

## Introduction

Nonsyndromic cleft lip with or without cleft palate (nsCL/P) is among the most common of all human birth defects (1). The genetic components of the underlying multifactorial etiology have been investigated extensively, with substantial recent progress due mainly to advances in large-scale genotyping technologies. Using genome-wide association studies (GWAS) together with replication assays, candidate gene and linkage studies, 20 genetic risk loci have been identified to date (2–11). A substantial fraction of these loci confer their effects in diverse populations (10–14), although the strength of the associations for single variants in different populations varies as a function of differences in risk allele frequencies and locus heterogeneity; also reflected by different prevalence rates for nsCL/P observed in different populations (1,11,15). Despite these successes it remains unclear how much of the variance in nsCL/P risk can be explained by the risk loci identified to date or common genetic variation in general, and the identification of additional genetic factors contributing to nsCL/P is to be expected.

In addition, one interesting question is the extent to which genetic factors overlap between nsCL/P and isolated (nonsyndromic) cleft palate where the lip is unaffected (nsCPO); the second most common type of orofacial clefting (OFC), after nsCL/P (15). There is ongoing discussion as to whether both disorders represent different OFC subphenotypes with shared genetic factors or whether they are genetically distinct conditions. The latter hypothesis is supported by epidemiological studies showing larger recurrence risks in either of the nsCL/P and nsCPO groups respectively, with cross-over phenotypes showing recurrence risks only slightly above the population background rates (16–18). Moreover, while GWAS have identified numerous associations reaching genome-wide significance for nsCL/P, it was only recently the first common variant reaching genome-wide significance for nsCPO was identified (19,20). Notably, this missense variant in grainyhead-like 3 (*GRHL3* [MIM 608317]) did not show any association with nsCL/P (19). Despite these observations, candidate gene approaches have revealed variants nominally associated with both nsCL/P and nsCPO, the most conclusive being markers at the *FOXE1* locus (8,21), suggesting that some regions might contribute to both traits with small effect sizes. However, it has remained unclear whether or not shared polygenic components might be involved in risk for both malformations.

Given the recent advances in sequence and haplotype annotation of numerous human populations by projects such as 1000 Genomes (22) or Genomes of the Netherlands (23), improved imputation and statistical methods as well as increasingly large sample sizes, it has now become possible to generate and comprehensively analyze high-density imputed datasets for complex phenotypes including nsCL/P and nsCPO. Such datasets have the power to identify novel associations that had escaped prior detection due to insufficient coverage of that region and/or low linkage disequilibrium (LD) between the genotyped markers and causal variants. In addition, this approach harbours enormous potential for the identification of allelic heterogeneity between populations and (sub-)phenotypes, and overlap with functionally annotated regions. Therefore, in the present study, we used data from previously published GWAS datasets for both nsCL/P (2,7) and nsCPO (24) from European and Asian populations, part of which we obtained from dbGaP with approved data access, to increase our understanding of the genetic architecture. Our results provide systematic novel insights in both genetic etiology and underlying biology of both types of facial malformation.

## Results

### Genome-wide imputation in nsCL/P and meta-analysis (discovery phase)

We used genotypes from our previously published meta-analysis of nsCL/P (6) which comprised 399 cases, 1318 controls (Bonn GWAS cohort (7)) and 1461 case-parent trios (Baltimore study: 666 European, 795 Asian (2), Supplementary Material, Fig. S1). After imputation and quality control (QC), 8.01 million variants were retained in the analysis. The genomic inflation factor was 1.044 for the analysis of European individuals only (meta_Euro_), and 1.047 for the overall analysis including Europeans and Asians (meta_all_). Quantile-quantile (QQ)-plots including the imputed data were similar when compared to QQ-plots including genotyped variants only (Supplementary Material, Fig. S2), indicating the absence of a technical bias due to the imputation performed. A total of 2556 SNPs showed *P*-values of at most 1×10^−^^05^ in at least one of the analyses, 2002 of which mapped to 17 of the 20 nsCL/P risk loci that were known at the time of analysis (no variants mapped to the *FOXE1* region and the chromosomal regions 17q23 and 19q13). Of the remaining 554 variants, two (rs6740960, rs4952552; both at chromosome 2p21) reached *P*_meta_Euro_ < 5×10^−^^08^ in the European analysis, while three additional variants at two loci met this threshold when Asian samples were included (meta_all_, Supplementary Material, Figs. S1 and S3, Table S1). These variants included two at chromosome 14q22 (rs4901118, rs60454187) and rs115681412 which maps to the major histocompatibility complex (MHC) region at chromosome 6. To exclude imputation errors at the highly variable MHC region (25), we performed a specific HLA imputation using T1DGC reference data, yielding *P* = 4.61 × 10^−^^08^ for rs115681412 in meta_all_. Notably, none of these five variants was present on the 550k/610k arrays used in the original studies.

The lead SNP at the newly identified risk region at chromosome 2p21 (abbreviated as 2p21_PKDCC_), rs6740960, maps 1.36 Mb away from rs7590268, the lead SNP located within the *THADA* gene at the previously identified locus within chromosome 2p21 (2p21_THADA_, (6)). We used conditional analyses in the Bonn case-control GWAS data to show that both effects are independent from one another: the *P*-value for rs6740960 (*P*_uncond._= 1.38 × 10^−^^04^, *P*_cond_rs7590268 _=_ _3.01 × 10^−^^04^) increased only marginally when conditioned on rs7590268, and also for rs7590268 (*P*_uncond._=1.68 × 10^−^^05^) when conditioned on rs6749060 (*P*_cond_rs6740960 _=_ _3.62 × 10^−^^05^).

### Replication and combined analysis

To confirm the findings obtained by imputation and in an attempt to identify further risk loci, 44 SNPs were selected for replication in an independent case-control cohort of mixed ethnicities (574 cases, 1635 controls, Supplementary Material, Fig. S1). Of the 42 SNPs passing QC, five showed *P *<* *0.05 (Supplementary Material, Table S3), including those at chromosomes 2p21_PKDCC_ and 14q22. No additional variant reached genome-wide significance when replication and imputed data were combined (Table 1, Supplementary Material, Table S1). Accounting for potential ethnic-specific factors, we additionally combined meta_Euro_ plus the European part of the replication cohort (i.e. the Bonn replication sample (*n* = 224 cases/921 controls); Supplementary Material, Table S1 and Fig. S1). In that analysis a missense variant at chromosome 19p13 (rs3746101; *MKNK2*, p.Q10K) reached genome-wide significance (*P *=* *2.44 × 10^−^^08^, Table 1). This variant was significantly associated with nsCL/P in the Central European (Bonn) component of the replication sample only (*P *=* *0.03) while it did not show association in either Mexican or Yemeni samples (*P *>* *0.3, Supplementary Material, Table S1).

**Table 1:** Association results for novel nsCL/P risk loci detected by imputation and replication

Locus	Lead SNP^a^	Chromosomal position	Alleles^b^	GWAS_imputed		*P*_Replication	Combined analysis	
				*P*_meta_Euro_	*P*_meta_all_		*P*_comb__Euro	*P*_comb__All
(a) Loci identified with genome-wide significance by imputation and replication								
2p21	rs6740960	42181679	**T**/A	3.29E-09	1.68E-09	3.32E-04	**1.86E-10**	**5.71E-13**
14q22	rs4901118	51856109	**A**/G	7.51E-07	5.44E-09	2.56E-02	2.00E-07	**6.94E-10**
19p13	rs3746101	2050823	**T**/G	2.30E-07	4.52E-04	3.17E-02 ^c^	**2.44E-08**	8.32E-05
(b) Loci identified with genome-wide significance by combining imputed Bonn GWAS dataset and (11) summary statistics								
15q24	rs28689146	75005575	**A**/T	6.54E-03 (3.6E-02)^d^	2.35E-02	5.4E-08^e^	n.a.^f^	**6.61E-09**

^a^Defined as lowest *P*-value in genome-wide imputation.

^b^Risk allele in bold.

^c^*P*-value for Bonn replication cohort only.

^d^Number in brackets indicates *P*-value for rs28689146 in Bonn GWAS case-control cohort. This cohort was used for in *silico* combination with replication cohort of Leslie *et al.* (2016) as there were overlapping individuals between Leslie *et al.* (11) and Beaty *et al.* (2).

^e^*P*-value from Leslie *et al.* 2016 (11).

^f^No summary statistics for rs28689146 in Leslie *et al.* (11) for the European dataset.

**Figure 1:**
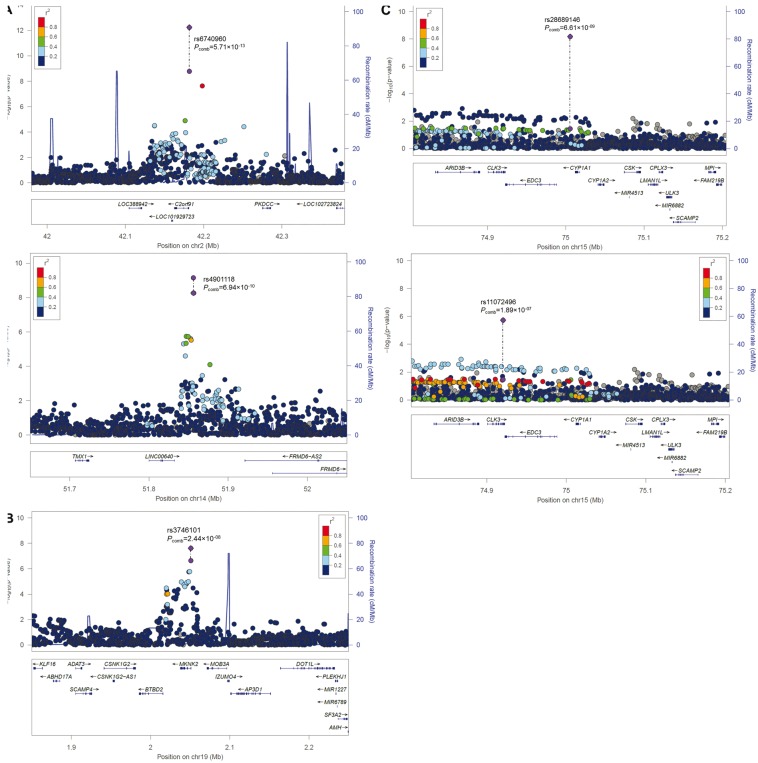
Regional association plots of four novel risk loci. Using genome-wide imputed data, *in vitro* and *in silico* replications, four novel risk loci for nsCL/P were identified. Plotted SNPs include both genotyped and imputed variants, respectively. (**A**) Two regions on chromosomes 2p21 and 14q22 reached genome-wide significance in the imputation analysis of meta_all_ and further decreased in *P*-values when replication data from a mixed ethnicity case-control cohort were added. (**B**) Analysis of meta_Euro_ and the European replication cohort revealed genome-wide significance for a region on chromosome 19p13. (**C**) *In silico* replication of previously suggestive findings at chromosome 15q24 (11) using the meta_all_ data revealed genome-wide significance for rs28689146. A second variant (rs11072496, bottom panel) fails to reach this threshold but improves capture of the haplotype structure. Plots were generated using LocusZoom (26).

The high-density dataset also allowed us to combine results directly with summary statistics available from other studies on nsCL/P without the need to use proxy SNPs. We assessed published data on 227 SNPs from a recent multiethnic study on nsCL/P (11), and combined these data with the imputed Bonn GWAS data (meta-analysis of the entire dataset was not possible due to individuals overlapping between studies (2) and (11)). In the combined analysis of European individuals (Supplementary Material, Table S4), no novel associations were detected. In the multiethnic combination (Supplementary Material, Table S5), we observed one locus at chromosome 15q24, previously reported as suggestive (11), which reached genome-wide significance (rs28689146, *P *=* *6.61 × 10^−^^09^, Table 1, Fig. 1).

### Associations with other traits

The present dataset on nsCL/P allows further analysis of related and/or non-disease traits *in silico*. For instance, it has been shown that unaffected family members of patients with nsCL/P show differences in facial morphometrics such as a broader nasal cavity, shorter upper and longer lower faces (27), and seven genetic loci have been found to be associated with various craniofacial morphometric traits (28–30). Analyzing these variants in our dataset did not reveal any significant association (*P *>* *0.2, Supplementary Material, Table S6), nor did any variants at plus/minus 500 kb withstand correction for multiple testing. We also searched for yet unknown trait associations with nsCL/P risk regions by assessing the GWAS catalogue (31) using both the list of credible SNPs and all SNPs in strong linkage disequilibrium (i.e. *r*^2 ^>^ ^0.8) in the European population. Three SNPs at two loci were associated with two non-clefting traits, i.e. the *FOXE1* locus with thyroid cancer (32), and the chromosome 3p11 locus with ocular axial length, a quantitative measure for myopia (33) (Supplementary Material, Table S7). At the *FOXE1* locus, the risk allele of two variants mapping close to a previously identified craniofacial enhancer hsCNE + 22.6 (34) showed decreased risk for thyroid cancer (32). In addition, two common variants located within this particular enhancer (rs12342417, rs10984103) showed association with risk for nsCL/P and are listed among the credible SNPs (Supplementary Material, Table S8, see next paragraph).

### Identification of credible SNPs

We next aimed at identifying SNPs which are highly probable to have a causative contribution to nsCL/P, i.e. the “credible SNP” set, for each of the 24 risk loci reaching genome-wide significance in our present study (*n* = 4) and previous research (*n* = 20, Supplementary Material, Table S9, (2–11)). We herefore used a Bayesian framework analysis in the meta_Euro_ dataset (Table 2, Supplementary Material, Table S8). In five of the nsCL/P regions, including chromosome 8q24, the set of credible SNPs did not encompass the genotyped lead SNP identified in the respective GWAS where these loci were originally identified. For two regions, at chromosomes 2p21_PKDCC_ and 17q13, only one SNP had a posterior probability of 95%, suggesting these were causative in the European population.

**Table 2:** Analysis of credible SNPs at 24 nsCL/P risk regions

Lead SNP	Closest gene	Chr	N SNPs	Length of region (bp)	SNP region 95% posterior probability				SNP region 99% posterior probability				References	Genotyped top SNP included^a^
					*N*	Start	End	Interval (bp)	*N*	Start	End	Interval (bp)		
rs742071	PAX7	1	317	110514	21	18939509	18992553	53044	33	18925512	18992583	67071	Ludwig *et al.* (6)	Yes
rs560426	ABCA4	1	124	41257	6	94539386	94551450	12064	9	94539386	94570218	30832	Beaty *et al.* (2)	No
rs642961	IRF6_DIEXF	1	1373	584708	18	209950760	210022893	72133	25	209950760	210022893	72133	Rahimov *et al.* (9)	Yes
rs4441471	AC104623.2	2	536	210141	68	16706771	16735432	28661	77	16706771	16735432	28661	Leslie *et al.* (11)	–
rs6740960	C2orf91	2	758	174921	1	42181679	42181679	0	2	42181679	42198217	16538	*Present study*	–
rs7590268	THADA	2	1036	406228	9	43501585	43670144	168559	31	43453086	43839244	386158	Ludwig *et al.* (6)	Yes
rs7632427	EPHA3	3	1902	843457	158	89273019	89640350	367331	238	89273019	89751353	478334	Ludwig *et al.* (6)	Yes
rs1384062	FILIP1L_CMSS1	3	1222	722547	75	99436424	100088929	652505	175	99428998	100097616	668618	Beaty *et al.* (3)	Yes
rs12543318	DCAF4L2	8	1446	546749	2	88868340	88887382	19042	2	88868340	88887382	19042	Ludwig *et al.* (6)	Yes
rs987525	LINC00976	8	953	318710	3	129964873	129990382	25509	4	129958384	129990382	31998	Birnbaum *et al.* (4)	No
rs3758249	FOXE1	9	716	290739	88	100596439	100667871	71432	123	100585506	100670272	84766	Moreno *et al.* (8)	Yes
rs4752028	KIAA1598	10	466	365794	23	118786985	118890006	103021	28	118786985	118890006	103021	Mangold *et al.* (7)	Yes
rs8001641	LINC01080_SPRY2	13	498	167219	6	80697449	80701485	4036	11	80691352	80701485	10133	Ludwig *et al.* (6)	No
rs4901118	RP11-255G12.2	14	476	134026	2	51856109	51856566	457	2	51856109	51856566	457	*Present study*	–
rs1258763	GREM1_FMN1	15	482	99614	13	33043284	33054523	11239	19	33043284	33054523	11239	Ludwig *et al.* (5)	Yes
rs1873147	TPM1	15	385	101368	13	63311425	63314519	3094	14	63311425	63314519	3094	Ludwig *et al.* (6)	Yes
rs57490152^b^	CSK	15	1721	818259	287	74686398	75231234	544836	748	74632243	75446488	814245	*Present study*	–
rs8049367	CREBBP_ADCY9	16	217	43706	22	3968115	3982627	14512	33	3963187	3997415	34228	Sun *et al.* (10)	Yes
rs1880646	NTN1	17	245	77824	1	8919415	8919415	0	1	8919415	8919415	0	Beaty *et al.* (3)	No
rs227731	NOG	17	215	87850	4	54773238	54777585	4347	5	54770864	54777585	6721	Mangold *et al.* (7)	Yes
rs8071332^a^	TANC2	17	1079	618627	46	61043405	61389844	346439	73	61043405	61405359	361954	Leslie *et al.* (11)	–
rs3746101	MKNK2	19	208	74094	4	2048281	2051261	2980	10	2038819	2051261	12442	*Present study*	–
rs8113265	SLC7A9	19	1410	365251	185	33132098	33495231	363133	691	33132098	33497141	365043	Leslie *et al.* (11)	–
rs13041247	MAFB	20	390	146327	41	39238736	39286786	48050	62	39238736	39286786	48050	Beaty *et al.* (2)	Yes

*N*—number of SNPs in the SNP region interval.

^a^This column refers to whether the originally presented lead SNP from the respective GWAS (i.e. genotyped SNP), is among the credible SNP set. In studies marked by ‘–’, imputation had already been performed.

^b^Identified with lowest *P*-value in meta_Euro_ dataset.

### Insights into nsCL/P biology by annotating risk loci

To gain insights into the biology of nsCL/P, we next performed in-depth analyses of the 24 recognized risk loci. These approaches included DEPICT analyses to identify enriched gene sets and pathways, as well as conditional analyses to investigate potential secondary effects.

To identify tissues in which genes near all 24 nsCL/P associated SNPs are highly expressed, and gene sets showing significant enrichment among these risk loci, we used DEPICT (35) in two settings; (i) including the 24 risk loci (Supplementary Material, Tables S10 and S11), and (ii) all autosomal SNPs with *P *<* *10^−^^05^ in meta_Euro_ (Supplementary Material, Tables S10 and S11). In the tissue enrichment analysis, strong results were observed for tissues related to the stomatognathic system. The result for the MEsH term "dentition" met the false discovery rate (FDR) threshold. At the pathway level, our analysis did not identify any significantly enriched gene set that survived correction for multiple testing (at FDR < 0.05).

We also investigated potential secondary effects at each of the 24 risk loci, by performing conditional analyses on the respective lead SNP (i.e. the SNP with the lowest *P*-value except for the regions at chromosomes 2p21_PKDCC_ and 17q13 where the credible SNP with 95% posterior probability was chosen) using the imputed data from the Bonn GWAS cohort. This approach identified 64 SNPs with *P*_cond _<_ _10^−^^03^ and a lower *P*-value in the conditional analysis as compared to the un-conditioned situation (Supplementary Material, Table S14). The majority of these SNPs mapped to five of the 24 chromosomal risk loci (1p36, 2p24, 13q31, 17p13 and 19q13, Supplementary Material, Fig. S4).

### Co-localization of association signals in relevant regulatory regions

In contrast to many common phenotypes for which human material is available and has been used by projects such as Epigenomics Roadmap (36), follow-up analyses of genetic findings for nsCL/P are hampered by the early embryonic time-point of facial development where defects occur; and the lack of appropriate human tissue. We therefore annotated the high-density nsCL/P dataset with ChIP-seq data previously generated to map active enhancers in human neural crest cells (hNCC, (37)) and active promoters and enhancers in mouse craniofacial embryonic tissue (38). Since hNCC are the precursors of the mesenchymal progenitors that will go on to generate most of the facial cartilage and bone during craniofacial development, we hypothesized the previously generated ChIP-seq datasets in hNCC (i.e. p300, H3K4me1, H3K27ac and TFAP2A) could be re-analyzed to define a broader set of cis-regulatory elements, including primed enhancers (see Methods). To identify whether nsCL/P association signals from the genome-wide dataset are significantly overrepresented within regulatory regions considered active during craniofacial development, we performed co-localization analyses (Table 3). Similar *cis*-regulatory maps generated from mouse embryonic limbs (39) and IRF6 binding sites identified in human adult keratinocytes (40) were used as controls. We identified highly significant enrichments in hNCC when either active enhancers alone or a broader set of active and primed enhancers were considered. Similarly, significant overrepresentations were also seen with mouse craniofacial enhancers (Table 3). Lower levels of significance were observed with mouse craniofacial promoter regions, while no enrichment was found in the control datasets of mouse limb-tissue and human adult keratinocytes.

**Table 3:** Co-localization analyses of nsCL/P imputation analyses results in autosomal regions of functionally annotated datasets

Study name	Annotation	Number regions	Number SNPs	Enrichment meta_Euro_^a^		Enrichment meta_all_		References
				*P* groups	*P* nominal	*P* groups	*P* nominal	
Neural crest cell line (NCC)	Active enhancer	4293	6886	*<0.0001*	0.0473	*<0.0001*	*<0.0001*	Rada-Iglesias *et al.* (37)
Mouse craniofacial tissue E11.5 (MCT_P)	Active promoter	4250	15 297	0.002	0.03	*0.0001*	0.041	Attanasio *et al.* (38)
Mouse craniofacial tissue E11.5 (MCT_E)	Active enhancer	7068	24 743	*0.0002*	*0.0002*	*<0.0001*	0.004	Attanasio *et al.* (38)
Neural crest cell line (Active and primed CRMs)	Active and primed enhancers	22 792	180 810	*<0.0001*	*<0.0001*	*<0.0001*	*<0.0001*	Data from Rada-Iglesias *et al.* (37) reanalyzed
Neural crest cell line (Active CRMs)	Active enhancers	16 177	141 231	*<0.0001*	*<0.0001*	*<0.0001*	*<0.0001*	Data from Rada-Iglesias *et al.* (37) reanalyzed
Mouse limb tissue E11.5 (CON1)	Active enhancer	2030	3713	0.10	0.18	0.30	0.35	Visel *et al.* (39)
IRF6 binding sites in keratinocytes (CON2)	IRF6 binding sites	3893	1219	0.6	0.26	0.005	0.19	Botti *et al.* (40)

CON—control datasets, CRM—*cis*-regulatory module.

^a^*P*-values one-sided; *italics* if significant after correction for multiple testing (*P *<* *0.00156).

### Estimation of variance explained in nsCL/P

**Figure 2:**
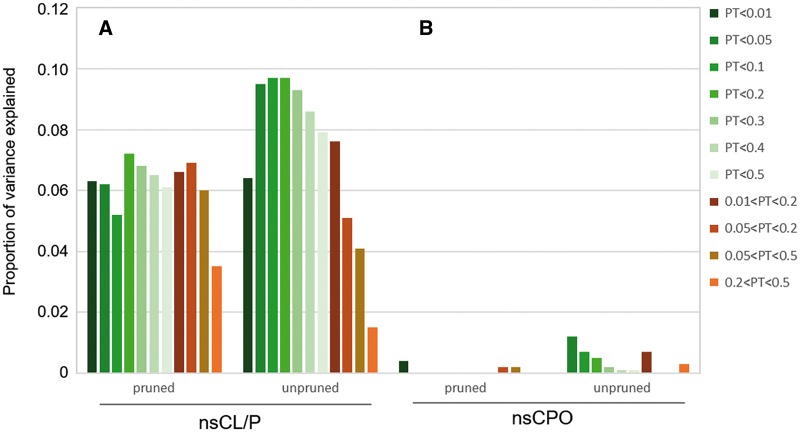
Polygenic risk score analysis in nsCL/P and nsCPO. The amount of variance explained in the target samples based on the polygenic score derived from the Bonn case-control GWAS nsCL/P discovery sample: (**A**) for the nsCL/P European trio sample; (**B**) for the nsCPO European trio sample. The amount of variance explained, denoted as Nagelkerke’s pseudo *R*^2^, is shown on the y-axis. Different association test *P*-value thresholds (*P*_T_) in the discovery dataset are represented by different colours. Results are shown for both pruned and unpruned analyses, respectively.

We estimated the heritability explained by common variants in the imputed Bonn GWAS dataset using the software GCTA (41). In addition, we estimated the proportion of variance in risk for nsCL/P that can be attributed to the recognized 24 risk loci using logistic regression. We found that 32.1% (8.5% standard error) of variance in the GWAS data can be attributed to common risk factors, with 25.5% contributed by these 24 risk regions. We also determined to what extent a polygenic component contributes to nsCL/P. We generated score alleles in the Bonn case-control GWAS cohort (discovery sample) and tested them for enrichment in the nsCL/P target sample (i.e. Baltimore trio cohort of European descent). We observed a highly significant enrichment of score alleles at a variety of *P*-value thresholds (Supplementary Material, Table S15A), with the lowest *P*-value in the Wald test (*P*_Wald_) being obtained for the unpruned matched SNP set at *P*_T_ < 0.2 (*P*_cWald_ = 2.04 × 10^−^^11^) with an estimated *R*^2^ of 9.7% (Fig. 2). The amount of explained variance was observed to be slightly higher for the unpruned dataset as compared to the pruned dataset (maximum *R*^2 ^=^ ^6.8%).

### Insights into the genetic etiology of nsCL/P and nsCPO

To investigate the potential etiological overlap between nsCL/P and nsCPO, we generated and analyzed imputed data from a previous genome-wide nsCPO study that included 550 nsCPO trios ((24), see Methods). No variant reached the threshold of genome-wide significance in either the European or in the analysis of mixed ethnicities (Supplementary Material, Figs. S5 and S6). The only genome-wide significant risk locus for nsCPO identified so far (19, 20) had *P*_nsCPO _=_ _1.18 × 10^−^^04^.

The lead SNPs and associated regions of the 24 recognized nsCL/P risk loci were analyzed in the nsCPO dataset (Supplementary Material, Table S16). At lead SNP level, only rs3758249 at the *FOXE1* locus showed significant associations in nsCPO after correction for multiple testing (*P*_nsCPO_all _=_ _1.84 × 10^−^^03^), which confirmed prior findings on associations with both traits (8,21). This finding was supported by additional variants being strongly associated at this locus (Supplementary Material, Fig. S7). Three other nsCL/P lead variants (at chromosomes 15q22, 16p13 and 17q22) were significant at the nominal level (Supplementary Material, Table S16). To investigate allelic or haplotype heterogeneity between both traits, the 24 nsCL/P loci (defined as nsCL/P lead SNP ± 500 kb) were extracted from the nsCPO data, revealing 73 078 SNPs passing QC. Fourteen variants had *P*-values lower than 10^−^^04^ in the overall analysis (Supplementary Material, Table S17), eight of which mapped to the 2p21 (*THADA*) region previously identified (6). The lowest *P*-value was observed for rs12476091 (*P*_nsCPO_all _=_ _8.55 × 10^−^^07^) which approaches the threshold of significance (conservative Bonferroni correction for number of SNPs, i.e. 6.84 × 10^−^^07^). Comparison of the regional association plots revealed two distinct association clusters for nsCPO and nsCL/P. Interestingly, conditional analyses in nsCL/P revealed suggestive association for variants overlapping the nsCPO-associated region as secondary effect (Supplementary Material, Fig. S8).

To address a polygenic contribution between nsCL/P and nsCPO, we finally sought to systematically analyze (1) the joint contribution of the 24 recognized risk loci, and (2) a general polygenic risk score from nsCL/P applied to nsCPO, using genome-wide data. For the first approach we generated dosage values for transmitted alleles and non-transmitted alleles in each of the nsCPO trios, for each of the 24 nsCL/P lead SNPs (Supplementary Material, Table S8). In each trio, both dosages were weighted with the logarithmic relative risks (RR) of the nsCL/P risk allele and summed over all 24 lead SNPs. This approach resulted in a transmitted and non-transmitted score for each nsCPO trio. Subtracting the non-transmitted score from the transmitted score revealed a mean over families of −0.1351 ± 1.51, indicating that in aggregate, these 24 recognized lead SNPs for nsCL/P do not contribute to nsCPO risk. The difference from a mean of 0 was not significant (*P *=* *0.195).

For the second approach, we used the polygenic score generated in the nsCL/P discovery dataset and evaluated its association in the nsCPO dataset of European ancestry trios (Supplementary Material, Table S15B). In none of the settings (pruned, unpruned, matched and non-matched) was a significant enrichment of nsCL/P risk allele scores in nsCPO detected (*P *>* *0.1, Supplementary Material, Table S15, Fig. 2).

## Discussion

In the first wave of GWAS, analysis of common variants was limited to a subset of ∼500 000 variants present on commercially available microarrays. Although these variants had been selected to tag the majority of genetic variation, it has become evident that true associations can be missed in situations where risk variants had a minor allele frequency lower than 5%; were not adequately tagged by the genotyped variants; or failed genotyping for technical reasons. With advances in the fields of high marker-density population reference sets and bioinformatic techniques, accurate prediction of non-genotyped variants has enabled further discoveries of the genetics underlying complex disorders (42–44).

Here, we performed large-scale imputation analyses of previously published GWAS datasets on nsCL/P and nsCPO, two of the most common types of congenital craniofacial anomalies, to detect novel risk loci. We identified common variants at four new loci as risk factors for nsCL/P, while none was identified for nsCPO. The associations at the chromosomal regions 2p21_PKDCC,_ 14q22 and 19p13 were replicated by genotyping in independent samples and are therefore deemed as associated with ‘high confidence’, while chromosome 15q24 revealed genome-wide significance upon *in silico* combination of two GWAS datasets. Notably, one variant in the highly variable MHC region identified with genome-wide significance in the nsCL/P genome-wide dataset did not replicate in any of our replication samples, suggesting a false-positive result similar to previous findings (11). As with most of the previous GWAS hits in nsCL/P and other complex traits, the top associated variants at chromosomes 2p21_PKDCC_ and 14q22 map in non-coding regions, and might therefore represent regulatory mutations affecting adjacent genes. Among the candidate genes at these loci are the protein kinase domain containing, cytoplasmic gene, (*PKDCC* [MIM 614150]), whose orthologues are involved in facial development in both mice (45) and zebrafish (46), FERM-domain containing 6 (*FMRD6* [MIM 614555]) and thioredoxin-related transmembrane protein 1 (*TMX1* [MIM 610527]). In contrast, the top-associated variant at chromosome 19p13 is a missense variant in MAP Kinase Interacting Serine/Threonine Kinase 2 (*MKNK2* [MIM 605069]). Although modelling MKNK2 protein structure using Phyre2 (47) revealed considerable changes in protein folding (Supplementary Material, Fig. S9), functional annotation using the Variant Effect Predictor (VEP), as well as a low CADD-Phred score (2.72) suggest rs3746101 may be a benign polymorphism. These observations suggest the biological effect in cleft pathogenesis might also result from mis-regulation of either *MKNK2* or, more likely, any of the numerous adjacent genes. In the latter case, candidates at the chromosome 19p13 locus would be BTB domain containing (*BTBD2* [MIM 608531]) which has been suggested to be involved in early zebrafish development (48), or casein kinase gamma 2 (*CSNK1G2* [MIM 602214]) which is involved in Wnt/beta-catenin signaling during anterio-posterior patterning (49).

Our study increases the total number of conclusive common risk factors for nsCL/P to 24, and using credible SNP analyses in the comprehensive European data suggest likely causative SNPs at each of these loci. Notably, our results suggest rs6740960 (2p21_PKDCC_) and rs1880646 (17q13) as likely causative SNPs in European populations. For other regions, such as those at chromosomes 17q22 (*NOG*) and 1q32 (*IRF6*), the credible SNP set contained fewer than 20 variants but included SNPs such as rs227727 or rs642961, which have been previously shown to have some functional effect (9,50). It remains unclear whether the same credible SNP sets would be identified in non-European populations; therefore, analyses of other distinct genome-wide datasets (e.g. studies (10) or (11)) would be required. Such an approach would also be helpful to delineate causal variants at other loci such as the *EPHA3*-locus (chromosome 3p11, > 50 SNPs in credible SNP set) using distinct LD patterns in other populations.

We next sought to analyze whether risk alleles of nsCL/P in aggregate contribute to risk for nsCPO. The lack of large etiological overlap between these disorders has been suggested by epidemiological studies, and we now support this hypothesis by a polygenic score approach based on actual GWAS data. Interestingly, during preparation of this manuscript, Wen & Lu developed a novel statistical method to account for phenotypic heterogeneity between disease subtypes and, when applying it to nsOFC datasets, confirmed that nsCL/P and nsCPO have different genetic etiologies (51). While there is ample evidence for nsCL/P being a multifactorial trait, the situation remains less clear for nsCPO. It is possible that nsCPO results from common risk loci which have not yet been detected (maybe due to low effect sizes and/or inadequate sample numbers). Alternatively, there is also a strong possibility that some patients with nsCPO might, in fact, be syndromic cases lacking the usual accompanying signs/symptoms. In this context, we have recently identified truncating mutations in *GRHL3*, a gene underlying the autosomal dominant Van der Woude syndrome (VWS [MIM 119300], (52)), in four patients with apparent nsCPO (19).

On aggregate, these observations suggest that nsCPO is likely more often caused by rare or low-frequency variants, while nsCL/P seems to follow the pattern of complex traits in which numerous common variants in non-coding regions are associated with moderate effect sizes. We suggest about one-third of the variance in risk for nsCL/P can be explained by common variants in general, with one-quarter attributable to the 24 risk loci currently recognized. These findings suggest that further common variants are present in the nsCL/P dataset and will have to be identified by increasing sample sizes, clinical details and/or approaches to reduce the burden of multiple testing. Also, contributions of rare variants with modest to strong effect sizes, or gene-environment interactions will probably help to explain the remaining missing heritability. Based on this, subsequent analyses of the biological impact of common variants were performed for nsCL/P only.

Notably, using our dataset we were able to identify potential shared etiologies of nsCL/P. While association with hypothyroidism has been established at the *FOXE1* locus, the co-association of variants with myopia and clefting is a novel observation suggesting shared etiologies. Co-incidence of clefting defects and eye malformations has been reported previously, for instance in Stickler syndrome (53) and anophtalmia (54). Interestingly, the risk allele for nsCL/P decreases axial length, indicating that a delayed cell growth or disturbed cell migration might be an underlying shared biological trait of these malformations. Our attempt to replicate single variants identified in craniofacial morphometric studies did not prove successful, however it remains possible that SNPs associated with craniofacial anomalies might contribute in aggregate to nsCL/P risk. Along these lines, recent analysis of a combined score of nsCL/P SNPs tested for facial traits revealed association for nose-width-related measurements, although with little variance explained (30). This suggests that polygenic risk scores might be helpful for determining whether these two distinct clinical entities share some genetic background.

Despite the advances in our understanding of nsCL/P etiology resulting from published GWAS and the current study, a number of limitations should be considered. First, our study did not have sufficient power to identify very low-frequency or rare variants with confidence; this limits its application with respect to following up causative variants suggested by functional or candidate gene studies which are in these frequency ranges. For example, rs138557689 at the *FZD6* gene locus was recently shown to be a functionally-relevant variant in an African-American family (55). In our dataset, this variant was excluded due to its low frequency (<1%). Future imputation studies using combined reference panels such the Haplotype Reference Consortium will increase the accuracy and informativity of low frequency variants and provide the opportunity to identify causal variants in this frequency range for nsCL/P similar to recent successes in other traits such as bone mineral density (56).

One important unanswered question is the exact functional mechanisms by which the risk alleles contribute to risk of clefting. The most likely scenario is that associated variants reside in *cis*-regulatory modules (CRM) that control expression of their target genes in a tissue- and time-specific manner. Therefore, the comprehensive annotation of CRMs in tissues contributing to craniofacial and palate development using, for example, epigenomic approaches, would be highly desirable. Considering the moderate sequence conservation of many regulatory elements, CRM maps should be ideally generated in relevant human tissue, which is a major hurdle given the early developmental time-points at which relevant craniofacial tissue should be collected. Alternatively, CRM maps can be generated in human cell types relevant for craniofacial development (e.g. neural crest), which can be derived in large amounts from human embryonic stem cells (hESC) using *in vitro* differentiation protocols. Moreover, relevant *in vivo* material can be isolated from embryonic animal models. Chiefly, our approach of co-localizing strongly associated variants from the nsCL/P GWAS with available CRM maps from murine embryonic craniofacial tissues and hNCC, shows that associated variants are significantly more often located within regions with regulatory potential (e.g. enhancers) than expected by chance. Notably, this enrichment was not observed when control datasets were considered, illustrating the importance of using CRM maps from relevant tissues and developmental stages. Using previously generated epigenomic datasets in hNCC, we defined a broad set of CRMs which included enhancers predicted to display a primed state that precedes and probably facilitates the future activation of these enhancers and their target genes (57,58). Interestingly, this broad CRM map was highly enriched in nsCL/P association signals, suggesting genetic variation might lead to epigenetic alterations at relevant CRMs very early during human embryogenesis, which could then affect the regulatory networks controlling craniofacial development at later prenatal stages. Elucidating if these early epigenetic alterations actually take place and understanding their mechanistic basis might uncover novel causal relationships which are not yet fully understood. To overcome the limitations associated with the tissues and developmental stage specificity of CRMs, further cell types and tissues should be functionally annotated, which could certainly span the number of causative variants that can be identified and molecularly characterized.

In conclusion, our study generates novel insights into the etiology of nonsyndromic orofacial clefting by revealing novel common variants and biological information for nsCL/P and demonstrating its distinct genetic background from nsCPO. Based on this, further work will now focus on identifying the biological mechanisms by which the associated common variants interfere with normal craniofacial development. Joint consortia efforts such as FaceBase (59) and the development of novel technologies such as massively parallel regulatory assays (60, 61) might provide tremendous resources and opportunities in that direction.

## Materials and Methods

### Genome-wide imputation (including HLA) plus meta-analysis (*discovery phase*)

In 2012, our group performed the first meta-analysis on nsCL/P using an in-house case-control dataset (Bonn GWAS dataset (7)) and a trio dataset (Baltimore study (2), Supplementary Material, Fig. S1). Three-hundred-ninety-nine cases, 1318 controls and 1461 individuals had been included, and 497 084 observed SNPs were analyzed (6). In the present study, these genotype data from both case-control and trio cohorts were imputed using IMPUTE2 (62), using 1092 individuals from the 1000 genomes project (22) as reference panel. For statistical analysis in the case-control cohort, logistic regression was performed (with SNPTEST and -method expected (63)), by incorporating the first five dimensions of the multi-dimensional-scaling coordinates. For the trio data retrieved from dbGaP (2), a previously published method was used (FBATdosage (64)). *P*-values were subsequently combined using Z-scores, in two approaches: European individuals only (meta_Euro_), and European plus Asian individuals (meta_all_). Analysis of Asian data only was not covered by the dbGaP request. To account for the limited power of imputation approaches to correctly predict rare and low-frequency variants, we only retained in the analysis SNPs with a SNPTEST info_score > 0.4 in any of the two datasets (i.e. case-control/trio) and a minor allele frequency (MAF) > 1% in the controls and non-transmitting parental alleles.

For the MHC region, a HLA-specific imputation was performed. SNP2HLA (65) was used to impute SNPs, amino acid residues, indels, and two- and four-digit classical alleles for eight HLA genes in the MHC region from 29 to 33 Mb on chromosome 6p21.3. We used the reference panel provided by T1DGC, which included 5225 European samples with classical typing for eight HLA genes (66).

### nsCL/P replication study (*replication phase* and *combined analysis*)

The replication sample consisted of 610 nsCL/P cases and 1737 controls from three different cohorts (Bonn, Yemen, Mexico, Supplementary Material, Fig. S1). The same sample was previously used in an independent study, please refer to (5) for sample description. Forty-four SNPs were included in the replication assay. Genotyping was performed using Maldi-ToF on an in-house Agena Bioscience Sequenom platform. Two SNPs failed genotyping (rs6030889, rs3091552) and were excluded, leaving 42 SNPs in the analysis (Supplementary Material, Table S1). After genotyping, 138 individuals (36 cases, 102 controls) had to be excluded due to call rates <95%. Final sample numbers were: 224 cases/921 controls (Bonn), 152 cases/323 controls (Mexico) and 198 cases/391 controls (Yemen).

Association statistics were calculated by applying the Armitage-trend test, for each sample cohort separately. For each SNP, relative risks of the three replication cohorts were combined using fixed-effect meta-analysis. For the combination of these datasets with the imputed data of meta_Euro_ and meta_all_, Z-score based analyses were used.

### *In silico* replication: combination with Leslie *et al.* (2016) (11) using summary statistics

In a recent multiethnic GWAS, three novel risk loci for nsCL/P were identified (11). As there was considerable overlap between individuals (*n* = 317) used in this study (11) and those used in the Baltimore study (2) (i.e. which were part of the meta-analysis (6)), results of this study could not be added to the imputed meta-analysis. In an attempt to combine independent samples, we therefore combined the Bonn-case-control cohort with (a) data from the European analysis of (11), and (b) data from the multiethnic cohort of (11). Summary statistics (*P*-values, risk allele, relative risks and confidence intervals) were extracted from Table 1, Supplementary Tables S4 and S5 (11) and combined with imputed Bonn GWAS data generated herein. SNPs comprised either imputed SNPs with *P *<* *10^−^^07^ or genotyped SNPs that had yielded *P *<* *10^−^^05^ in (11). In the European dataset (comprising 406 trios, 170 cases and 835 controls), 102 SNPs met these quality criteria, 86 of which were also represented in the imputed dataset of the Bonn GWAS cohort. In the multiethnic cohort (comprising 1319 trios, 823 cases and 1700 controls), 238 variants were identified at these *P*-value thresholds, 227 of which were also present in the Bonn GWAS data. These data were combined using fixed-effect meta-analysis.

### Genome-wide imputation of nsCPO data set

Genotypes for 550 parent-child trios with nsCPO were retrieved from a previous genome-wide nsCPO study (24). Similar to nsCL/P, variants were imputed using 1000 genomes as reference panel, and statistical analysis was performed using FBATdosage (64) for about 8.38 million variants (info scores ≥0.4, MAF ≥ 1% in the non-transmitting parental alleles). Again, analysis was split into an European analysis only (CPO_Euro_), and an analysis of mixed ethnicities (CPO_all_).

### Polygenic score analysis

We performed polygenic score analysis (a) to analyze whether a polygenic component contributes to the genetic susceptibility of nsCL/P, and (b) to determine whether this polygenic component of nsCL/P contributes to risk for nsCPO. Based on (67), the polygenic score analysis was performed using the imputed Bonn GWAS data as the discovery cohort and two target cohorts, i.e., European nsCL/P trios from the Baltimore study (2) for analysis (a) as well as European nsCPO trios (24) for (b). We used SNPs with MAF ≥ 1% in the Bonn GWAS data and info-scores ≥0.8 in each of the target cohorts.

We generated 11 SNP sets using different thresholds from the association test *P*-value (*P*_T_) in the discovery sample. For each SNP set and each individual, a score in the target sample was calculated as the number of risk alleles which were weighted using the log relative risks obtained from the discovery sample. The polygenic score analysis was performed for all SNPs and also after pruning on linkage disequilibrium. The latter approach enabled analysis of a collective contribution of independent risk alleles (*r*^2 ^<^ ^0.25). These polygenic scores were tested to determine their association with disease status using logistic regression (unmatched) and conditional logistic regression (matched) which accounts for correlation status between cases and pseudo-controls. Nagelkerke’s pseudo *R*^2^ was used to estimate the proportion of the variance of risk to disease in (a) nsCL/P-trios and (b) nsCPO-trios explained by the polygenic risk score. The Wald test was used to test for the effect of the polygenic score on risk for nsCL/P and nsCPO.

### Co-localization analysis

The tissue-specific mode of action of regulatory elements and the very narrow developmental time range of cleft development (during 6^th^ to 8^th^ week post-conception) suggest that causative variants in nsCL/P could be identified by overlapping high-density genetic datasets with epigenetic datasets from relevant embryonic tissue or cell types. Given the limitations associated with embryonic tissue datasets from humans, we used publicly available datasets for both human neural crest cells (hNCC, (37)) and mouse facial tissue (38). In these datasets, active enhancer and promoters were identified based on different combinations of epigenetic marks. Moreover, we re-analyzed the ChIP-seq datasets previously generated in hNCC (37) to define broader sets of CRMs in which promoter regions were not explicitly excluded. These additional CRMs were expected to include primed enhancers that are inactive but pre-marked in hNCC and which might become active later in hNCC development, for example when differentiating into craniofacial mesenchyme. To define these larger CRM maps, hNCC ChIP-seq datasets were first re-analyzed with MACS2 (68) using the following criteria:

- p300 and TFAP2A peaks were identified using *q* ≤ 10^−4^.
- H3K4me1: peaks were identified using broad settings and *q* ≤ 10^−4^.
- H3K27ac: peaks were identified using broad settings and *q* ≤ 10^−6^.

Next, these peaks were analyzed in a combinatorial manner to define two sets of CRMs:

- *Active CRMs* (16 177 regions): regions bound by p300 or TFAP2A and enriched in H3K27ac within 1Kb.
- *Active and Primed CRMs* (22 792 regions): it included the Active CRMs defined above, plus regions enriched in both H3K27ac and H3K4me1.

In addition, two control cohorts containing similar datasets generated from mouse embryonic limbs (39) and IRF6 binding sites identified in human adult keratinocytes (40) were used.

For the co-localization analysis, two different categorization schemes for *P*-values were applied. The first scheme (*P* groups) consisted of the nine categories 10^−^^k + 1 ^≥^ ^*P *>* *10^−^^k^ for k = 1, …, 8 and 10^−^^8 ^≥^ ^^ ^*P*. The second schema (*P* nominal) only distinguished between *P *>* *0.05 and *P *≤* *0.05. For both schemes, the frequencies of SNPs being located within and outside annotated regions were counted within each category. The one-sided version of the Cochran-Armitage test for trend was applied to test for an enrichment of smaller *P*-values within annotated regions on the basis of the resulting 9×2 or 2×2 contingency table.

### Statistical refinement of genetic associations at 24 loci

A Bayesian refinement method was applied at each of the recognized risk loci to determine the subset of most informative SNPs, referred to as the credible SNP set (69). This approach has previously been demonstrated to be more efficient at selecting putatively functional SNPs compared to linkage disequilibrium based approaches (70,71). A genomic interval around each index SNP was defined by a genetic distance of 0.1 centimorgans (cM) upstream and downstream of each index SNP using HapMap fine-scale recombination rate estimates. An assumption of the Bayesian refinement method is that each locus contains a single association, therefore each interval was tested for multiple independent associations by including the index SNP as a covariate in the logistic regression and repeating the analysis conditional on this SNP. Where multiple associations exist the credible SNP set was calculated for one SNP while fixing the effect of the other by including it as a covariate in the logistic regression. To calculate the credible SNP set for each independent effect the posterior probability that any particular SNP is the casual SNP was calculated based on the Bayes factor for the SNP as a proportion of the mean Bayes factor for all SNPs in the genomic interval. Posterior probabilities were aggregated to define the smallest set of SNPs with a total posterior probability of  ≥95% and 99% (69).

### Estimation of variance explained

The GCTA (41) analysis of the explained variance in nsCL/P risk was performed using the imputed data of the Bonn case-control GWAS dataset. As GCTA requires genotypes as input, individual genotypes were called using a threshold of 90%. The resulting dataset was filtered by keeping SNPs with genotype call rates ≥ 95%, MAF ≥ 1%, HWE *P*-value ≥10^−^^06^ and non-significant differences in missingness between cases and controls (PLINK (72) *P*-value ≥ 0.05). In total, 5.61 million SNPs, both genotyped and imputed, respectively, remained after this quality control of the dataset. Using GCTA and a relationship cutoff of 5% estimated from the genome-wide markers, we obtained the Genetic Relationship Matrix (GRM) for 1711 individuals (i.e. 399 cases and 1318 controls). The same first five dimensions of the multi-dimensional-scaling coordinates as for the association analysis were included in the estimation of the explained variance by GCTA. A disease prevalence of 0.1% was assumed.

### DEPICT

We applied DEPICT (35) to systematically analyze most likely causal genes at the given associated loci, explore pathways enriched for nsCL/P associations and reveal tissues and cell types where genes from associated loci are highly expressed. The DEPICT framework relies on publicly available datasets and uses large and diverse gene expression data, allowing annotation of potential functional connections between genes at associated loci. Here, we downloaded the beta version of DEPICT_v1 (http://www.broadinstitute.org/mpg/depict/; date last accessed July 15, 2016) and performed the analysis in two settings: (i) all autosomal SNPs with *P *<* *5×10^−^^08^ plus three top SNPs for each of the known 24 loci that did not have genome-wide results in our analyses (n=816 SNPs); and (ii) all autosomal SNPs with *P *<* *5×10^−^^05^ in the meta_Euro_ data (n=1470 SNPs). The latter setting was chosen because limiting the analysis to known risk loci only may result in loss of power because SNPs that have not yet met appropriate thresholds of significance due to limited statistical power. In each setting, gene prioritization, gene set enrichment and tissue/cell type enrichment analyses respectively were performed.

## Supplementary Material

Supplementary Material is available at *HMG* online.

## Supplementary Material

Supplementary Data
