# Supplementary material for: Imputation of orofacial clefting data identifies novel risk loci and sheds light on the genetic background of cleft lip ± cleft palate and cleft palate only
Source: Hum Mol Genet. 2017 Jan 19;26(4):829–42. doi: 10.1093/hmg/ddx012 (PMC5409059; doi:10.1093/hmg/ddx012)

Supplementary Figure 1: Design of the present study

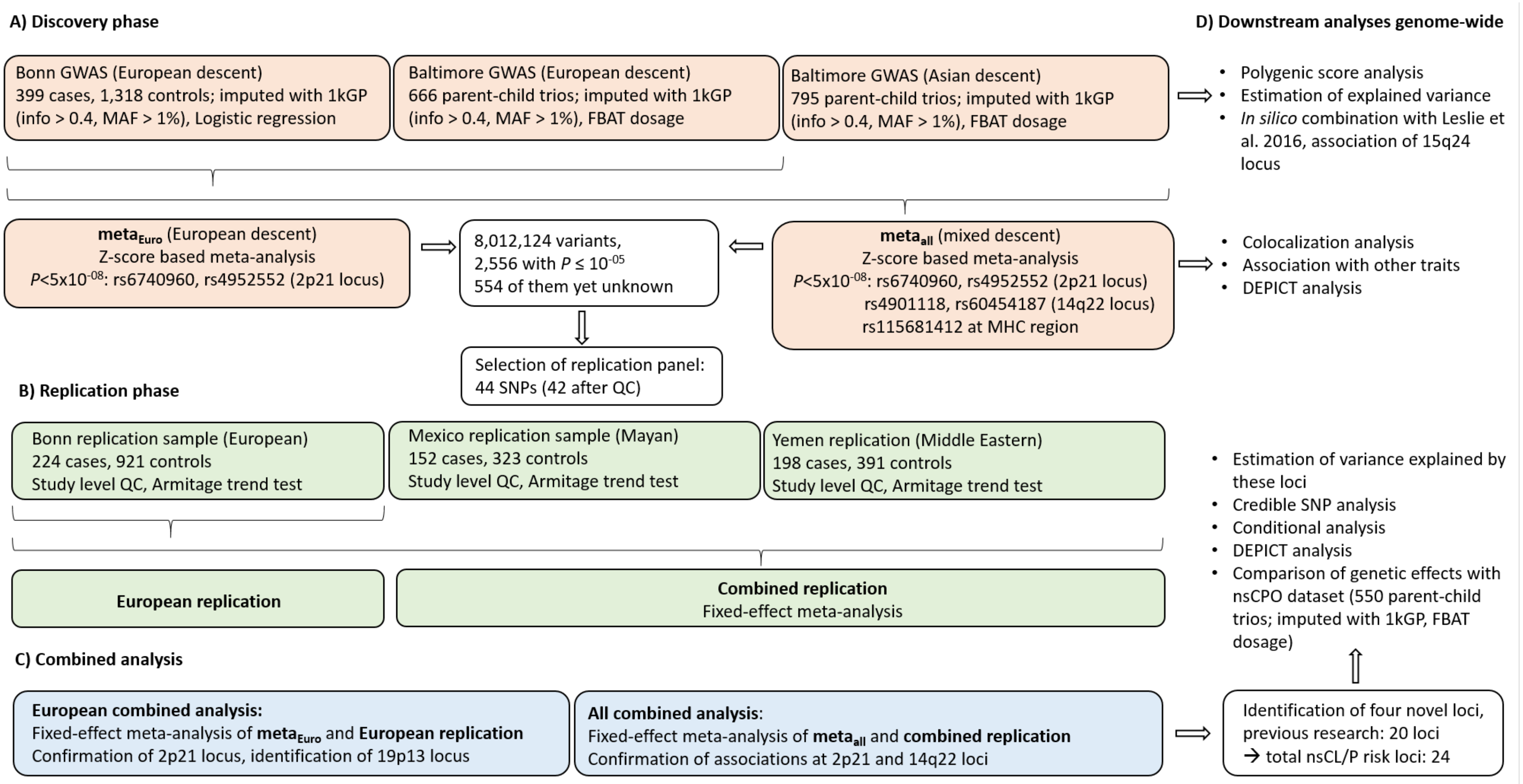

Supplementary Figure 2: Quantile-quantile plots for imputed data on nsCL/P

Quantile-quantile plot for 8,012,124 variants in the nsCL/P meta-analysis Euro (a, genomic inflation factor (GIF) = 1.044) and the nsCL/P meta-analysis all (b, GIF = 1.047). The y-axis was truncated at -log(*P*) = 8. Panels (c) and (d) refer to the genotyped SNPs of $meta_{Euro}$ and $meta_{all}$, respectively, indicating that the deviation from the null curve is not attributed to imputation artifacts but likely reflecting the biological component.

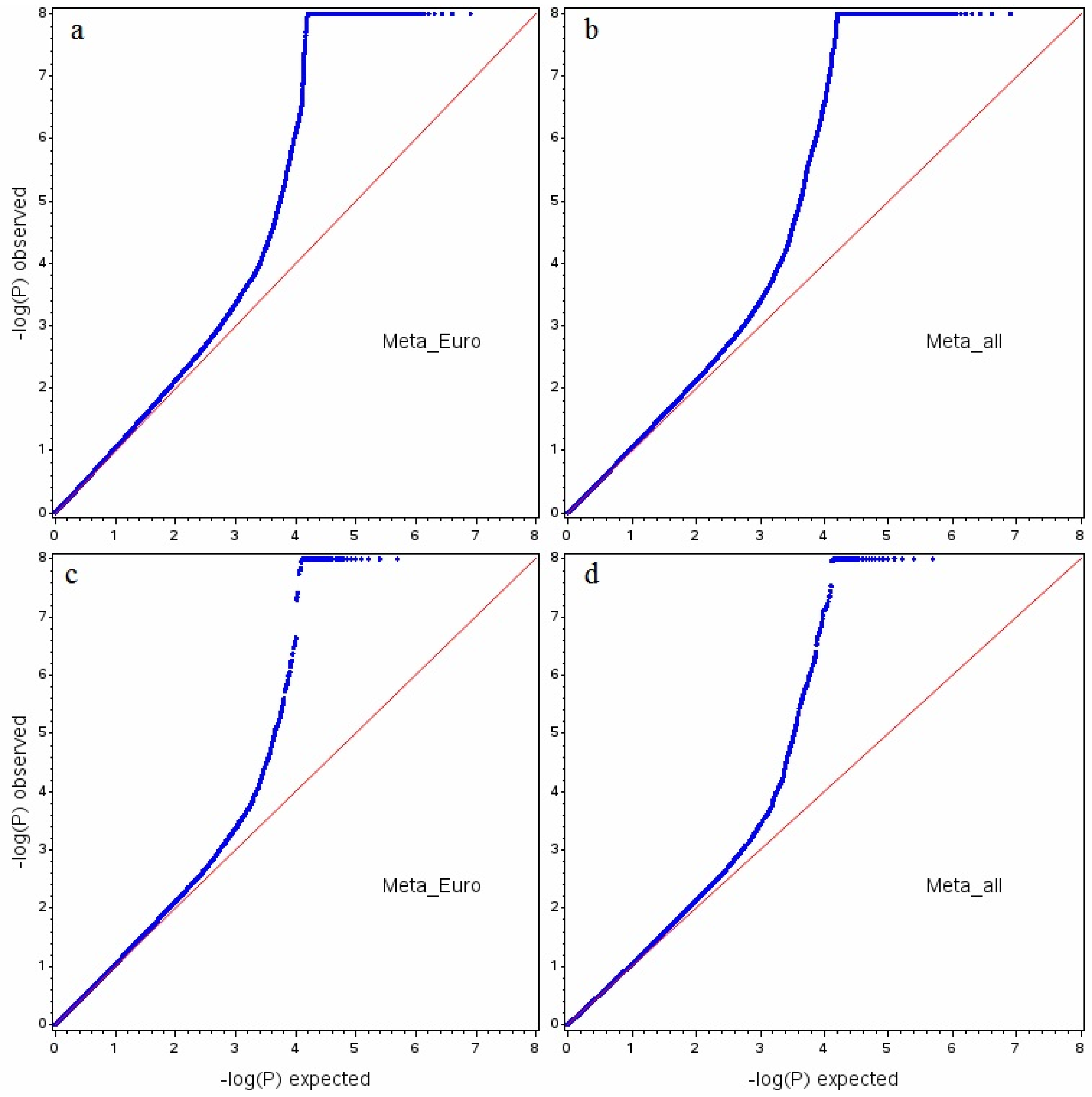

Supplementary Figure 3: Manhattan Plots for imputed data of nsCL/P.

In the top panel, the results of the impuation analysis in nsCL/P $meta_{Euro}$ are shown as association-log(P)-values plotted against chromosomal positions. The same plot is shown at the bottom, for $meta_{all}$.

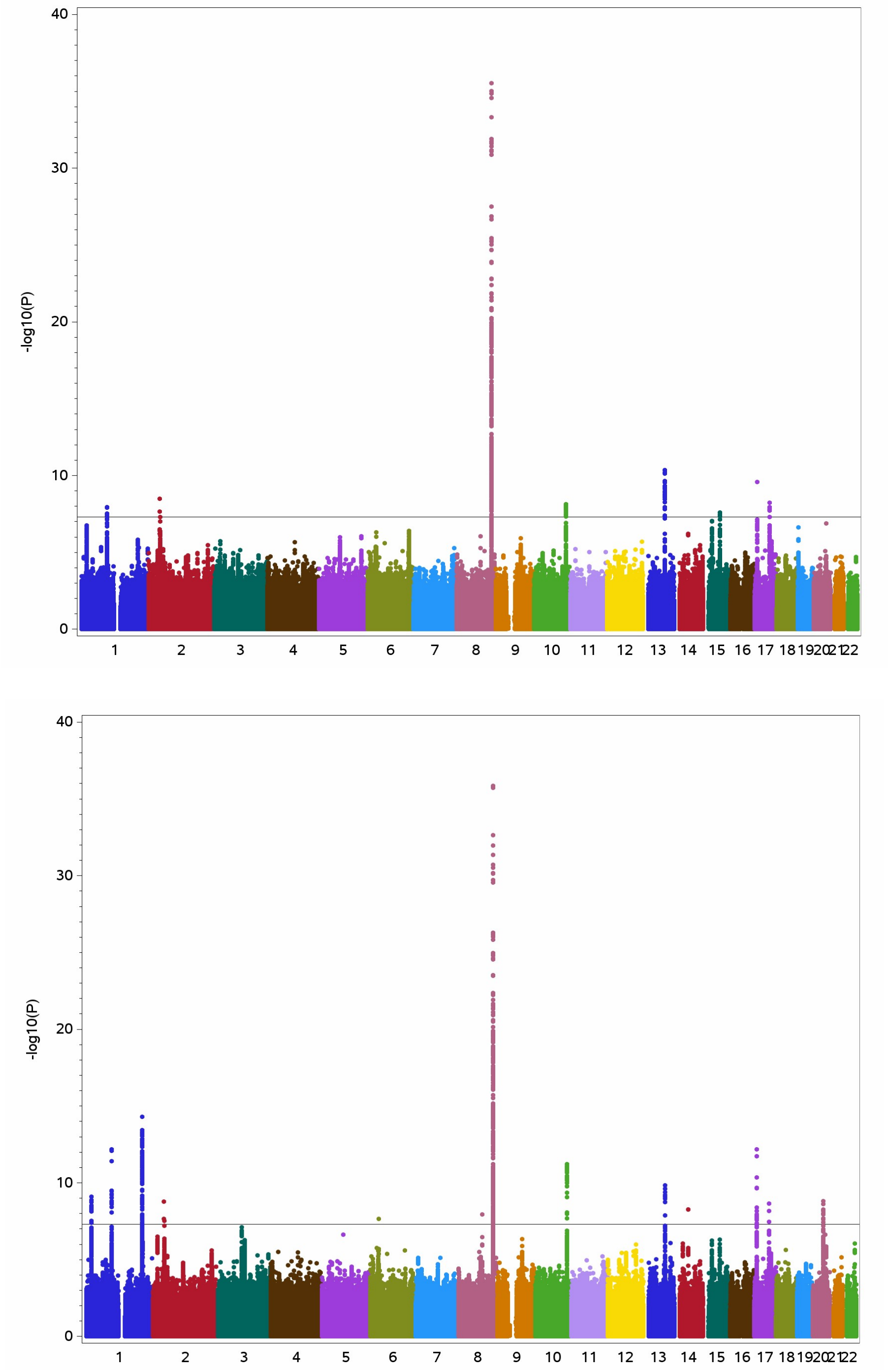

Supplementary Figure 4: **Analysis of secondary effects.**

Five of the 24 regions showed evidence for the presence of secondary effects as defined by *P*<0.001 and lower *P*-values in conditioned (lower panel) versus unconditioned (upper panel) states. For each region, the SNP that was used for the conditional analysis using logistic regression is indicated as lead SNP in the respective upper panel. For 17p13, the credible SNP (rs58772677) was used as lead SNP.

(a) 1p36

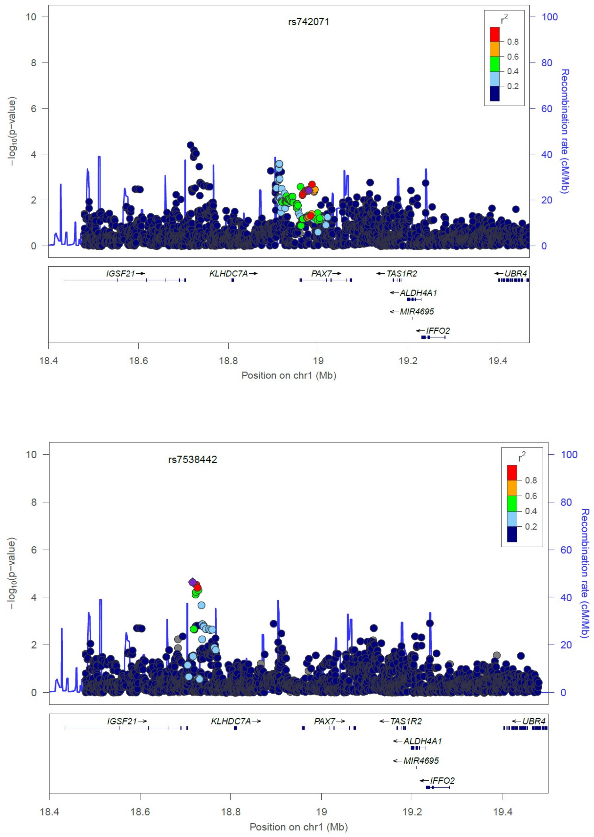

(b) 2p24

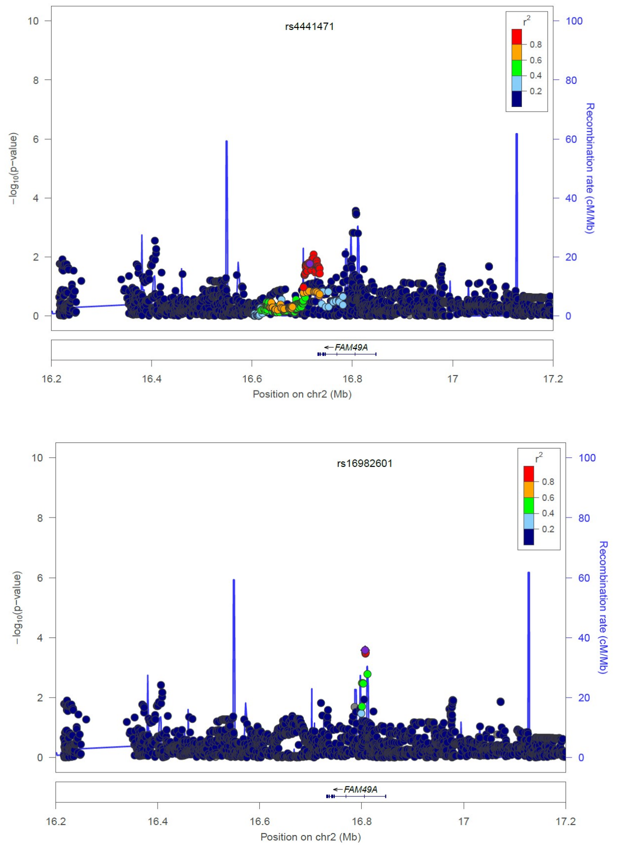

(c) 13q31

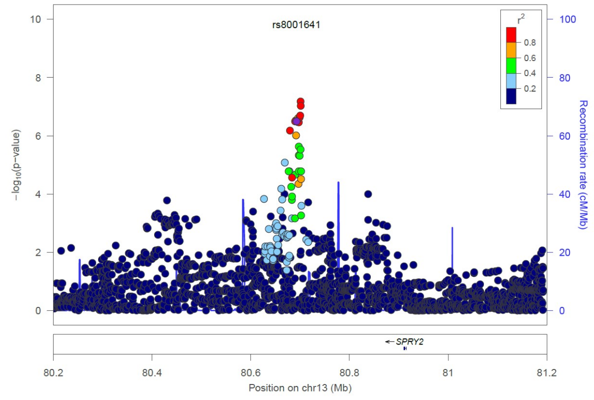

(d) 17p13

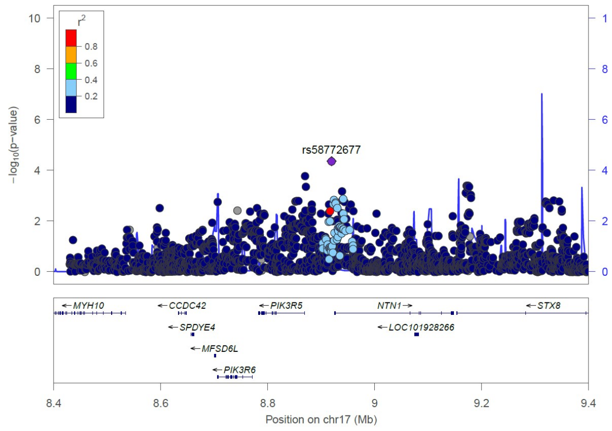

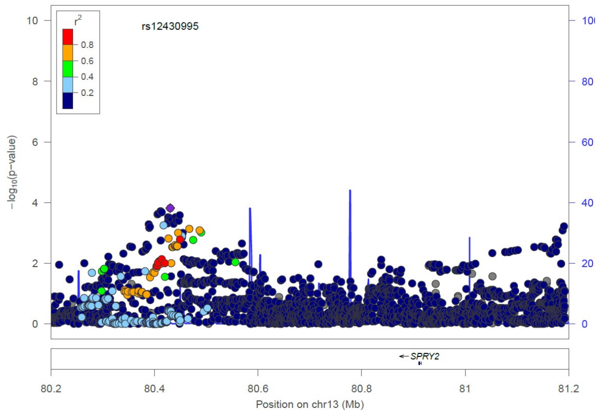
rs12430995
r2
0.8
0.6
0.4
0.2
−log10(p−value)
SPRY2
Position on chr13 (Mb)

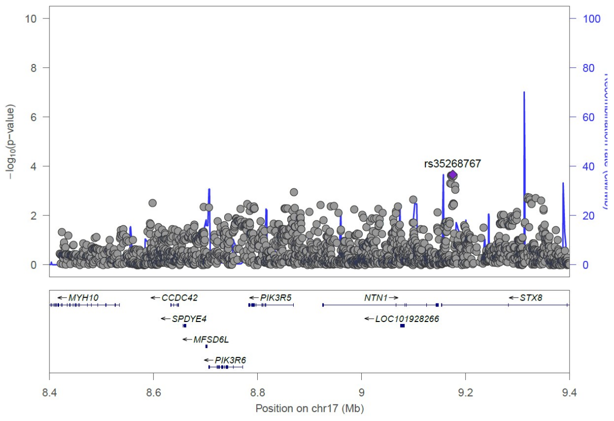
rs35268767
−log10(p−value)
MYH10
CCDC42
SPDYE4
MFSD6L
PIK3R6
PIK3R5
NTN1
LOC101928266
STX8
Position on chr17 (Mb)

(e) 19q13

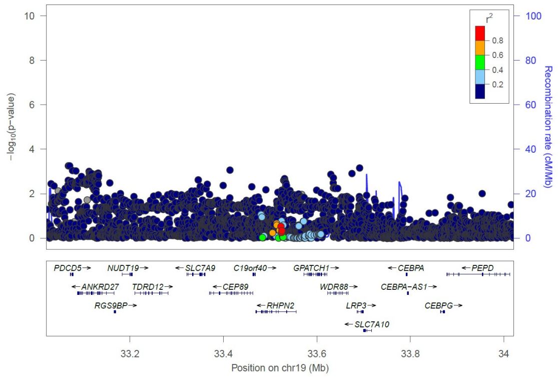
r2
0.8
0.6
0.4
0.2
−log10(p−value)
Recombination rate (cM/Mb)
PDCD5
NUDT19
SLC7A9
C19orf40
GPATCH1
CEBPA
PEPD
ANKRD27
TDRD12
CEP89
WDR88
CEBPA−AS1
RGS9BP
RHPN2
LRP3
CEBPG
SLC7A10
Position on chr19 (Mb)

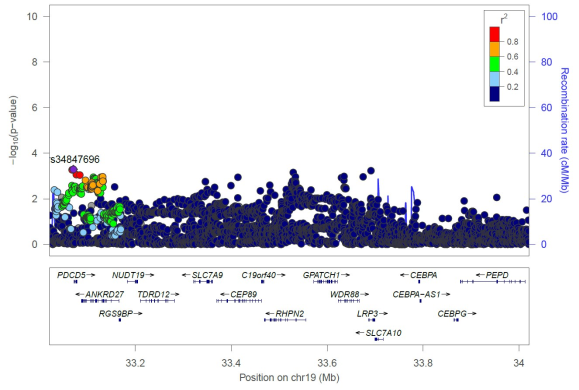
s34847696
r2
0.8
0.6
0.4
0.2
−log10(p−value)
Recombination rate (cM/Mb)
PDCD5
NUDT19
SLC7A9
C19orf40
GPATCH1
CEBPA
PEPD
ANKRD27
TDRD12
CEP89
WDR88
CEBPA−AS1
RGS9BP
RHPN2
LRP3
CEBPG
SLC7A10
Position on chr19 (Mb)

Supplementary Figure 5: Quantile-quantile plots for imputed data on nsCPO

Quantile-quantile plot for 8,384,634 variants in nsCPO Euro (top panel) and nsCPO all (bottom panel) analysis. Observed Association P-values (y-axis) are plotted as –log(P) versus expected values (x-axis).

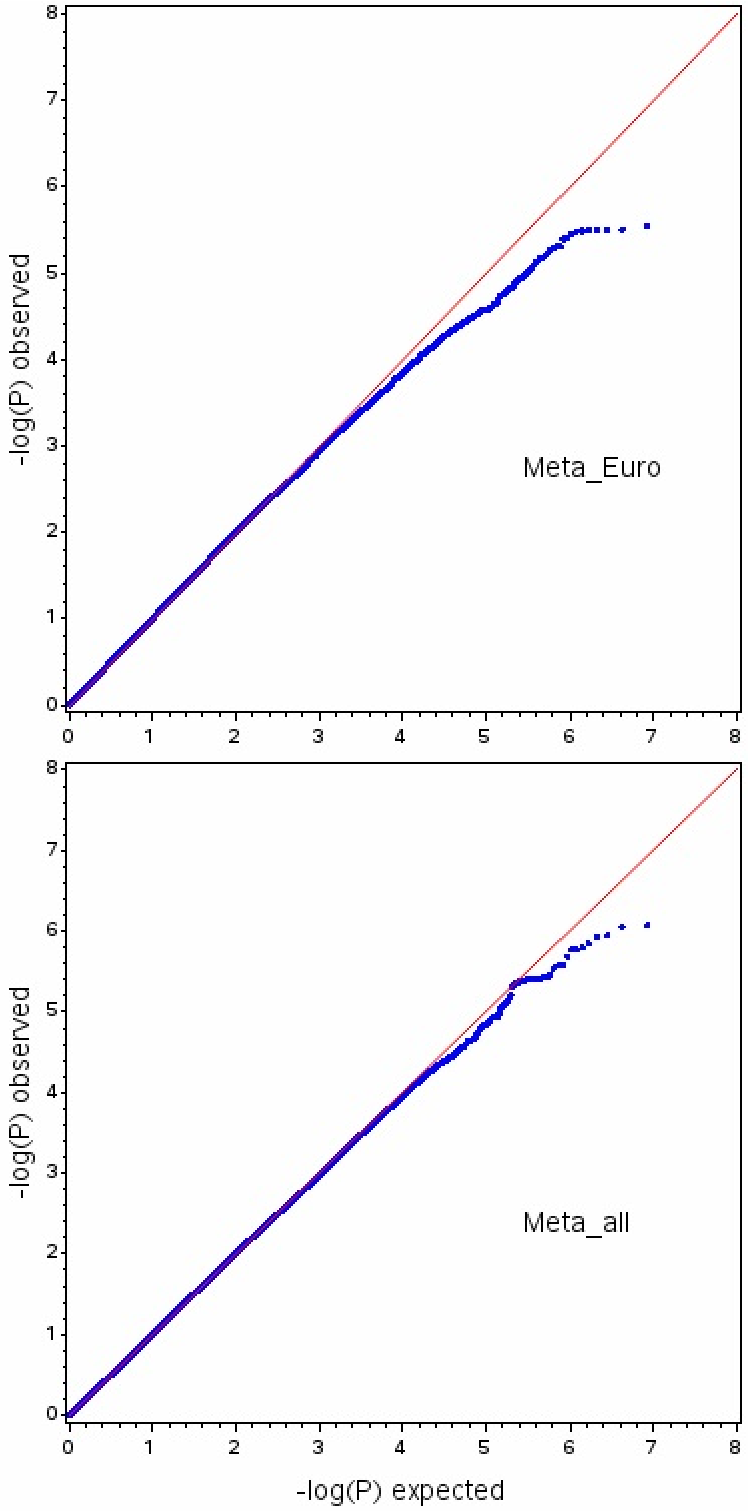

Supplementary Figure 6: **Manhattan Plots for imputed variants in nsCPO.**

In the top panel, the results of the imputation analysis in nsCPO_Euro are shown as association-log(P)-values plotted against chromosomal positions. The same plot is shown at the bottom, for nsCPO_all.

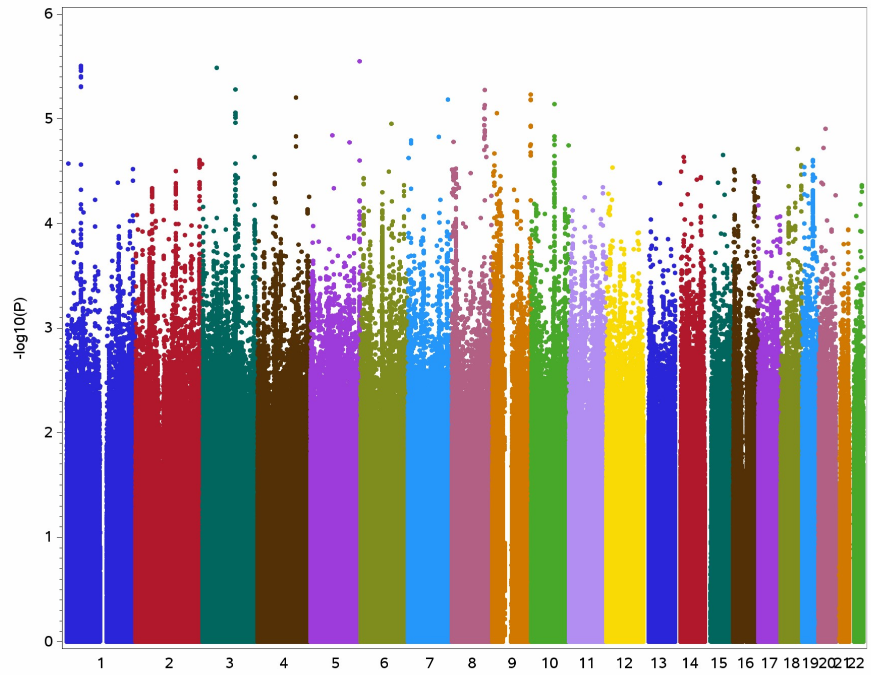

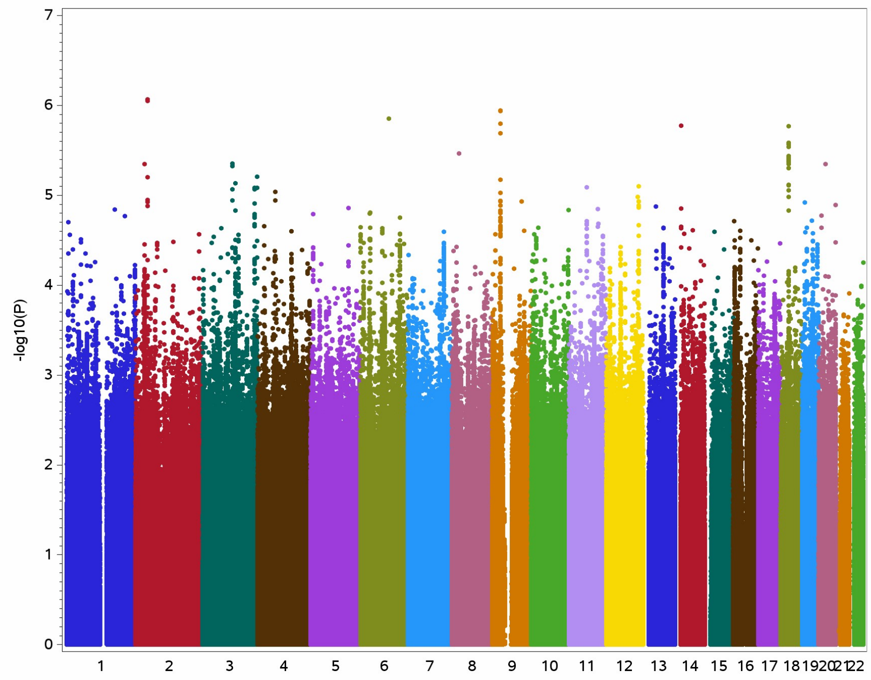

Supplementary Figure 7: Association of the *FOXE1* region with nsCPO.

We extracted a region plus/minus 500 kb around rs3758249 from the nsCPO data of European (**a**) and European-Asian (mixed) families (**b**). The top associated SNP in nsCPO, rs7045465 is located about 80 kb from rs3758249 and has considerable LD to it (r^2=0.43, D'=0.71, according to CEU 1000 genomes phase 1).

a

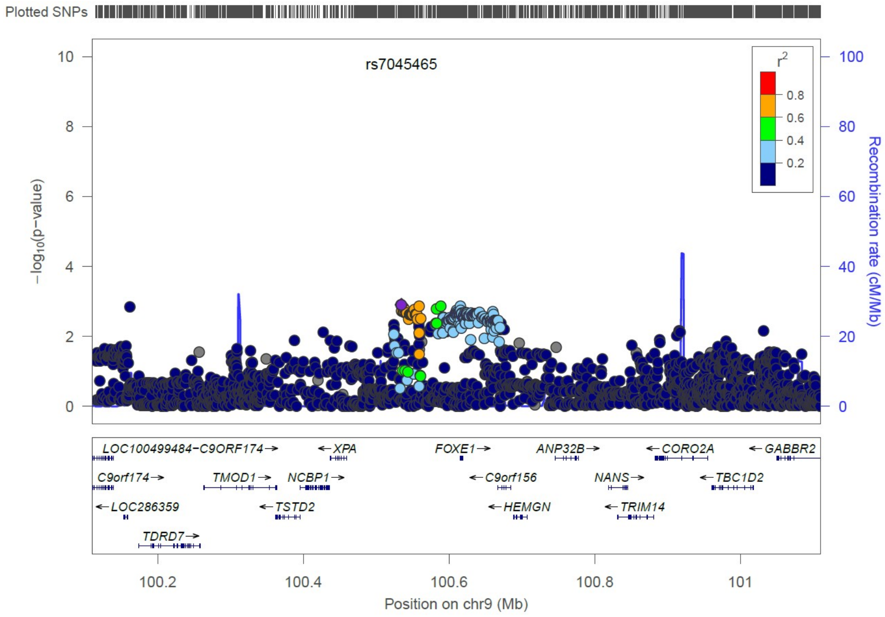

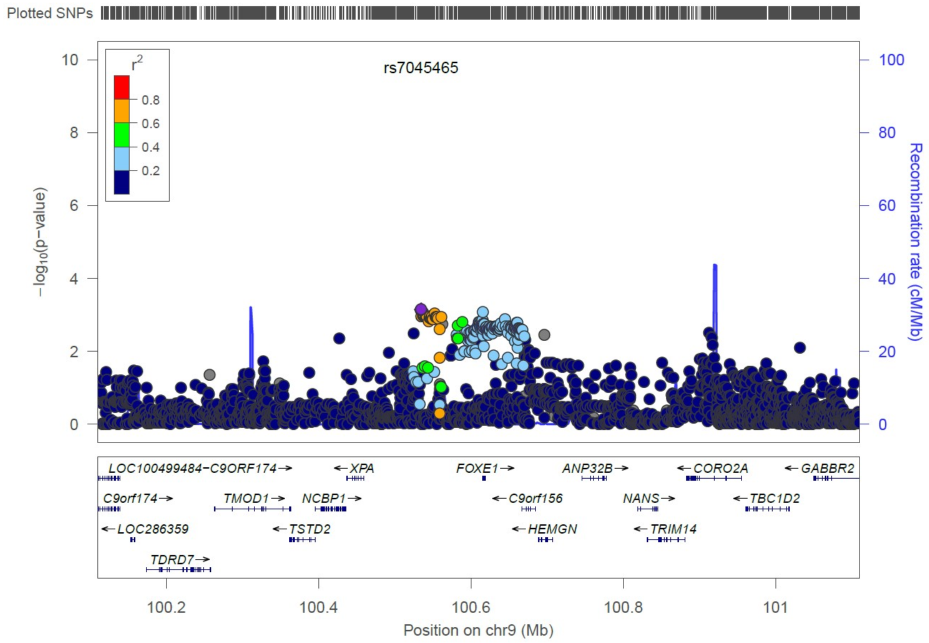

Supplementary Figure 8: Allelic heterogeneity between nsCL/P and nsCPO.

At the nsCL/P risk locus $2p21_{THADA}$, which was discovered in a previous meta-analysis (Ludwig et al. 2012), a distinct association signal is observed in nsCPO **(a)**, about 300 kb away from the primary nsCL/P risk region **(b)**. Notably, upon conditioning on rs7590268 in the Bonn GWAS case-control cohort **(c)**, a second independent hit in nsCL/P occurs that locates closely to the nsCPO-associated region.

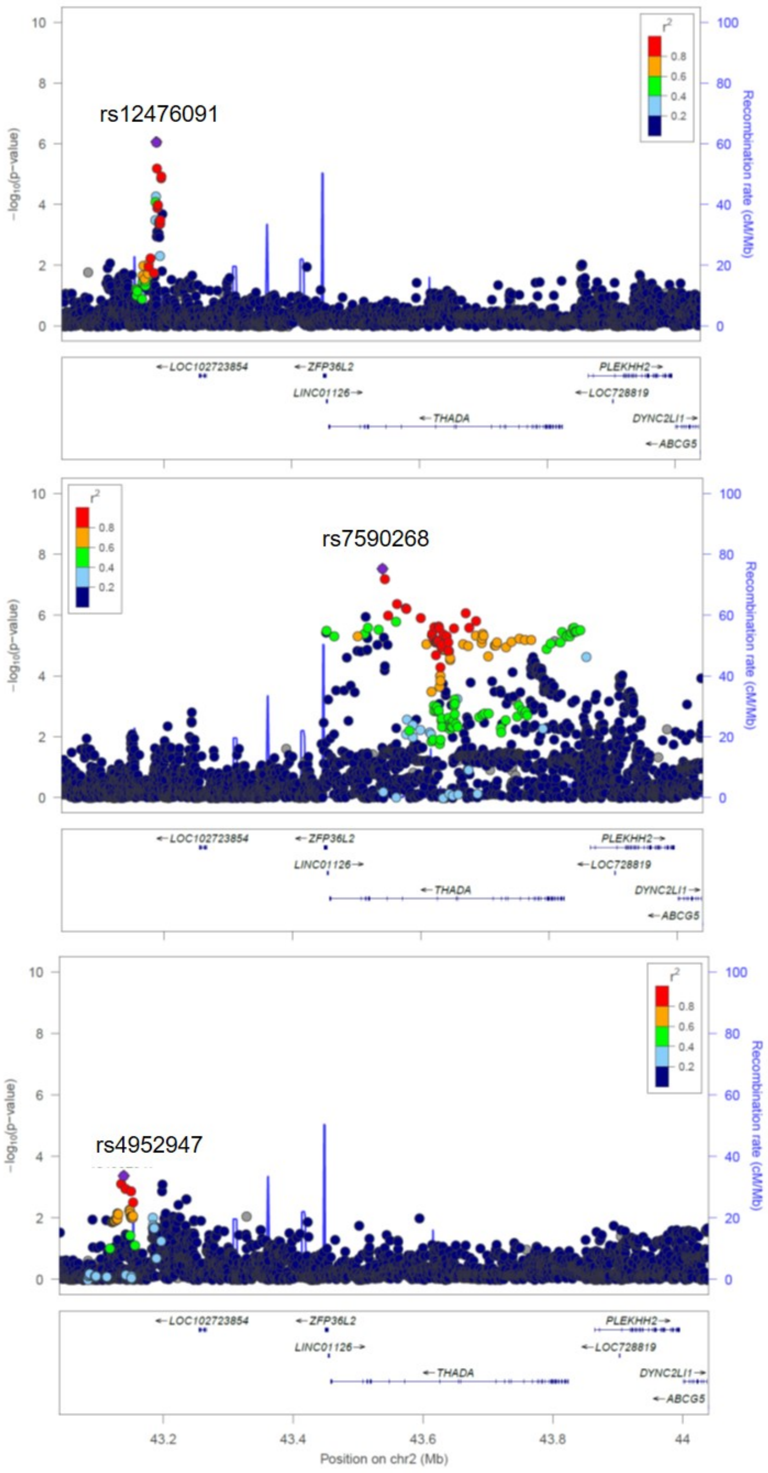

Supplementary Figure 9: Protein structure modelling of MKNK2 using Phyre2.

Secondary and tertiary structure of MKNK2 was modelled for both reference allele **(a)** and for the alternative allele **(b)** of SNP rs3746101, using the intensive mode of Phyre2. rs3746101 results in a missense variant (Q>K) in the protein at position 10 (p.Q10K). Tertiary protein models coloured by rainbow N->C. Please note different folding of the dark-blue and red regions in the variant version of the protein.

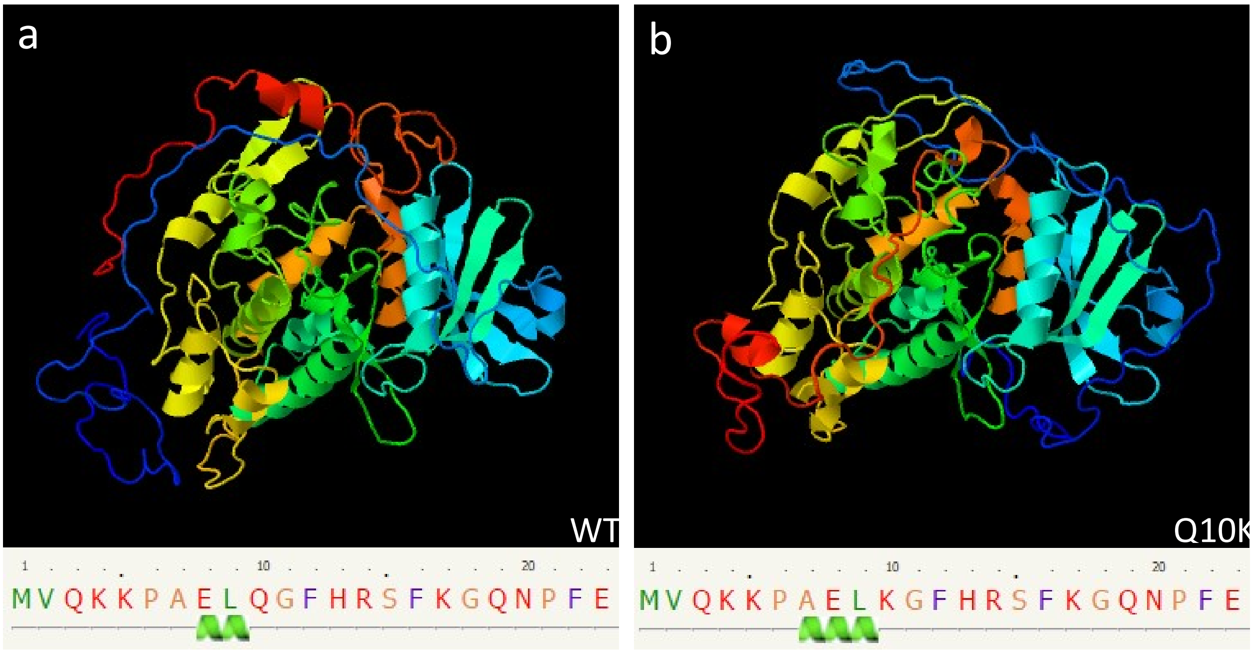

Supplement: Supplementary Data [file ddx012_Supp.zip › Supplemental_Figures_final.pdf]
