# Supplementary material for: Imputation of orofacial clefting data identifies novel risk loci and sheds light on the genetic background of cleft lip ± cleft palate and cleft palate only
Source: Hum Mol Genet. 2017 Jan 19;26(4):829–42. doi: 10.1093/hmg/ddx012 (PMC5409059; doi:10.1093/hmg/ddx012)
Supplement: Supplementary Data [file ddx012_Supp.zip › Supplemental_Text.docx]

**Additional acknowledgments for dbGaP accession** [**phs000094.v1.p1**](http://www.ncbi.nlm.nih.gov/projects/gap/cgi-bin/study.cgi?study_id=phs000094.v1.p1)**:**

Funding support for the dbGaP study was provided by several previous grants from the National Institute of Dental and Craniofacial Research (NIDCR). Data and samples were drawn from several studies awarded to members of this consortium. Funding to support original data collection, previous genotyping and analysis came from several sources to individual investigators. Funding for individual investigators include: R21-DE-013707 and R01-DE-014581 (Beaty); R37-DE-08559 and P50-DE-016215 (Murray, Marazita) and the Iowa Comprehensive Program to Investigate Craniofacial and Dental Anomalies (Murray); R01-DE-09886 (Marazita), R01-DE-012472 (Marazita), R01-DE-014677 (Marazita), R01-DE-016148 (Marazita), R21-DE-016930 (Marazita); R01-DE-013939 (Scott). Parts of this research were supported in part by the Intramural Research Program of the NIH, National Institute of Environmental Health Sciences (Wilcox, Lie). Additional recruitment was supported by the Smile Train Foundation for recruitment in China (Jabs, Beaty, Shi) and a grant from the Korean government (Jee). The genome-wide association study, also known the Cleft Consortium, is part of the Gene Environment Association Studies (GENEVA) program of the trans - NIH Genes, Environment and Health Initiative [GEI] supported by U01-DE-018993. Genotyping services were provided by the Center for Inherited Disease Research (CIDR). CIDR is fully funded through a federal contract from the National Institutes of Health (NIH) to The Johns Hopkins University, contract number HHSN268200782096C. Funds for genotyping were provided by the NIDCR through CIDR’s NIH contract. Assistance with genotype cleaning, as well as with general study coordination, was provided by the GENEVA Coordinating Center (U01-HG-004446) and by the National Center for Biotechnology Information (NCBI). We sincerely thank all of the patients and families at each recruitment site for participating in this study, and we gratefully acknowledge the invaluable assistance of clinical, field and laboratory staff who contributed to this effort over the years.
